# Isobolographic Analysis of the Cytoprotective Effect of Dapsone and Cannabidiol Alone or Combination upon Oxygen–Glucose Deprivation/Reoxygenation Model in SH-SY5Y Cells

**DOI:** 10.3390/antiox13060705

**Published:** 2024-06-08

**Authors:** Marcela Islas-Cortez, Camilo Ríos, Jorge Manzanares, Araceli Díaz-Ruiz, Ricardo Pérez-Pastén-Borja

**Affiliations:** 1 Laboratorio de Toxicología Molecular, Departamento de Farmacia, Escuela Nacional de Ciencias Biológicas, Instituto Politécnico Nacional, Ciudad de México 07738, Mexico; marc_omaet@hotmail.com; 2 Departamento de Neuroquímica, Instituto Nacional de Neurología y Neurocirugía “Manuel Velasco Suárez”, Ciudad de México 14269, Mexico; 3 Laboratorio de Neurofarmacología Molecular, Universidad Autónoma Metropolitana Xochimilco, Ciudad de México 04960, Mexico; lriosc@inr.gob.mx; 4 Dirección de Investigación, Instituto Nacional de Rehabilitación Luis Guillermo Ibarra Ibarra, Ciudad de México 14389, Mexico; 5 Instituto de Neurociencias, Universidad Miguel Hernandez-CSIC, 03550 San Juan de Alicante, Alicante, Spain; jmanzanares@umh.es; 6 Redes de Investigación Cooperativa Orientadas a Resultados en Salud (RICORS), Red de Investigación en Atención Primaria de Adicciones (RIAPAd), Instituto de Salud Carlos III, MICINN and FEDER, 28029 Madrid, Madrid, Spain; 7 Instituto de Investigación Sanitaria y Biomédica de Alicante (ISABIAL), 03010 Alicante, Alicante, Spain

**Keywords:** isobolographic analysis, dapsone, cannabidiol, oxygen–glucose deprivation and reoxigenation, oxidative stress, caspase-3 activity, synergistic effect

## Abstract

Oxidative stress and apoptosis cell death are critical secondary damage mechanisms that lead to losing neighboring healthy tissue after cerebral ischemia. This study aims to characterize the type of interaction between dapsone (DDS) and cannabidiol (CBD) and its cytoprotective effect in an in vitro model of oxygen and glucose deprivation for 6 h followed by 24 h of reoxygenation (OGD/R), using the SH-SY5Y cell line. For the combined concentrations, an isobolographic study was designed to determine the optimal concentration–response combinations. Cell viability was evaluated by measuring the lactate dehydrogenase (LDH) release and 3-[4, 5-dimethyl-2-thiazolyl]-2, 5-diphenyl-2H-tetrazolium bromide (MTT) assays. Also, the reactive oxygen species (ROS) and reduced glutathione (GSH) levels were analyzed as oxidative stress markers. Finally, caspase-3 activity was evaluated as a marker cell death by apoptosis. The results showed a decrease in cell viability, an increase in oxidant stress, and the activity of caspase-3 by the effect of OGD/R. Meanwhile, both DDS and CBD demonstrated antioxidant, antiapoptotic, and cytoprotective effects in a concentration–response manner. The isobolographic study indicated that the concentration of 2.5 µM of DDS plus 0.05 µM of CBD presented a synergistic effect so that in treatment, cell death due to OGD/R decreased. The findings indicate that DDS–CBD combined treatment may be a helpful therapy in cerebral ischemia with reperfusion.

## 1. Introduction

Cerebral ischemia is the leading cause of permanent disability, causing high social and economic costs; likewise, it is the third cause of death worldwide [1,2]. The interruption of blood flow in cerebral ischemia is the consequence of acute occlusion of a cerebral artery, such as the middle cerebral artery (MCA), leading to a lack of oxygen–glucose; it is hazardous for brain function, as it is an organ with high oxygen consumption, high metabolism, and low energy reserve [3,4]. The decrease in oxygen-glucose causes a sudden depletion of ATP, leading to a failure in the activity of the sodium/potassium pump, causing neuronal depolarization, increased release of glutamate, and increased calcium entry into the neuron. The caspases pathway is then activated, and in addition to the overproduction of reactive oxygen species (ROS), all of these events ultimately lead to neuronal death [5,6,7]. The mechanisms are still unclear; for this reason, the oxygen–glucose deprivation/reoxygenation (OGD/R) SH-SY5Y model is a tool to understand neuronal cell injury processes after hypoxia and hypoglycemia, providing us with an in vitro model to evaluate new treatments for cerebral ischemia. The lack of safe and effective neuroprotective therapies in clinical studies with ischemic stroke patients demonstrates the strong need to develop new strategies showing a clear functional benefit [8,9,10].

Dapsone has been described as a neuroprotective agent in cerebral ischemia in rats. The neuroprotective effect of DDS may be associated with its antioxidant capacity since the formation of ROS is prevented in many models of neuronal damage. Those findings are consistent with the reported actions of the drug, as several studies show that DDS diminishes oxygen radicals and decreases concentrations of hydrogen peroxide and hydroxyl radicals from oxidative activation of polymorphonuclear leukocytes [11].

In a model of vincristine-induced neuropathic nociception, the neuroprotective effect of DDS has been proposed to be a critical factor in the inhibition of myeloperoxidase and elastase, as well as the reduction of local production of ROS. Furthermore, DDS reduces oxidative stress by directly affecting the performance of neutrophils and monocytes. By activating the survival protein, brain-derived neurotrophic factor, and by inhibiting proapoptotic proteins such as c-Jun N-terminal kinase, phosphatidyl 3,4,5-trisphosphate 3-phosphatase, calpain, and caspase-3, it also reduces neuronal injury. These actions contribute to the protective function of DDS in neural tissue under ischemic conditions [12].

Likewise, there is evidence to suggest that cannabidiol (CBD) is an essential non-psychotropic phytocannabinoid that has therapeutic potential against different pathologies, including diseases associated with central nervous system (CNS) damage, such as ischemic stroke and other chronic diseases, including Parkinson’s and Alzheimer’s diseases [13,14]. There is also evidence showing that CBD decreases the release of glutamate and stabilizes the mitochondrial membrane [15,16]. Evidence for both drugs indicates that they may act as neuroprotective agents under in vitro and in vivo conditions of cerebral ischemia with reperfusion, suggesting that they might activate cooperative interaction processes to enhance neuroprotection.

However, the efficacy and therapeutic benefit when administered in combination and their interactions on the regulation of damage mechanisms after OGD/R are unknown. Our study aims to determine the degree of interaction by isobolographic analysis of co-administration in a cellular model of OGD/R using human neuroblastoma SH-SY5Y cells. Our main aim is to improve the therapeutic effect of both drugs when administered in an optimal combination.

## 2. Materials and Methods

### 2.1. Chemicals and Reagents

Cannabidiol (CBD) (dissolved in 0.01% DMSO, pure synthetic) was purchased from STI Pharmaceuticals (Essex, UK). Dapsone (DDS) (dissolved in 0.01% DMSO, purity > 97%) (1164008), dimethyl sulfoxide (DMSO) (472301), and 3-[4, 5-dimethyl-2-thiazolyl]-2, 5-diphenyl-2H-tetrazolium bromide (MTT) (M2128), O-phthalaldehyde (79760) were obtained from Sigma-Aldrich (St. Louis, MO, USA), also all-trans retinoic acid (RA) (R2625) (Sigma Aldrich, Milan, Italy). LDH assay kit (116447930 01) was from Roche (Mannheim, Germany). Additionally, 5-(and-6)-chloromethyl-2′,7′-dichlorodihydrofluorescein diacetate (CM-H2DCFDA) (C6827) was purchased from Molecular Probes, Eug, OR, USA, and the caspase-3 colorimetric kit (APT165) was from Merck Millipore (Burlington, MA, USA).

### 2.2. Cell Culture

The human neuroblastoma SH-SY5Y cell line was purchased from ATCC, Manassas, VA, USA. Cells were cultured in a 1:1 mixture of Dulbecco’s modified Eagle’s medium and Ham’s F12 medium (DMEM/F12) (Gibco-Thermoscientific, Grand Island, NY, USA) supplemented with 100 U/mL penicillin/streptomycin, 10% fetal bovine serum (FBS) certified (16000044) (Gibco-Thermoscientific, Grand Island, NY, USA) and 0.2% sodium bicarbonate (Sigma-Aldrich, St. Louis, MO, USA). The cells were maintained in an incubator at 37 °C in a saturated humidity atmosphere containing 95% air and 5% CO_2_; these conditions were used in all experiments unless otherwise stated. The culture medium was replaced every three days, and the cells were detached and subcultured at a ratio of 1:4 in standard culture medium and incubation conditions once they had achieved a confluence of no more than 90% through trypsin treatment. Before conducting any assays, the SH-5HSY cells were differentiated according to the protocol described by Teppola [17]. In summary, cells in suspension were seeded in microplates (dimension according to each experiment) at a concentration of 40,000 cells/well and standard culture medium supplemented with 10 µM all-trans retinoic acid (RA) (Sigma Aldrich, Milan, Italy) incubated in conditions described before for six days to induce differentiation. All experiments were performed in nine replicates per group.

### 2.3. MTT Cytotoxicity Assay

Cell viability was measured at the end of the exposure period using the 3-[4, 5-dimethyl-2-thiazolyl]-2, 5-diphenyl-2H-tetrazolium bromide (MTT) assay described by Martinez et al. [18], which consisted of replacing the culture medium with the treatments, with 200 μL of MTT dissolved in standard culture medium at a concentration of 1 mg/mL and incubating the microwell for 4 h in standard conditions. Finally, the medium containing MTT was withdrawn, leaving the well dry, and 200 μL of DMSO was added to each well to dissolve the purple formazan crystals. After 10 min, the absorbance of each well was measured using a microplate spectrophotometer (BioTek Eon) set to 490 nm. The following formulas were used to calculate cell viability: Cell viability (%) = cells in the experimental group divided by cells in the control group = 100% and % of Lethality = 100 − cell viability (%).

### 2.4. Mean Cytotoxic Concentration (LC_50_) Assay

The median lethal concentrations (LC_50_) of DDS and CBD were evaluated by subjecting SH-5HSY differentiated cells, which were cultivated in a 96-well microplate, to various doses ranging from 2.5 to 604 µM and 0.05 to 477 µM, respectively, for 24 h. The compounds were diluted in dimethyl sulfoxide (DMSO) to achieve a final concentration of 0.01% in the culture medium. DDS, CBD, and their combinations were diluted in all subsequent tests using the above method. After 24 h, the MTT method was employed to assess cell viability, which was subsequently quantified as a percentage relative to the viability of unexposed cells.

### 2.5. Oxygen–Glucose Deprivation/Reoxygenation (OGD/R) Model and Treatment

Culture under OGD/R conditions mimics the cellular conditions of cerebral ischemia and was used to assess DDS and CBD’s cytoprotective capacity. According to the protocol above, differentiated SH-SY5Y cells in 96-well microplates had the culture medium removed. After three washes with Hank’s buffered saline solution (HBSS), the HBSS was replaced with standard culture medium without glucose and incubated in a humidity-saturated anaerobic chamber with an atmosphere consisting of 5% CO_2_ balanced with N_2_ and tempered at 37 °C for 6 h. Under these conditions, cell viability decreased by 50%. Then, the glucose-free medium was replaced with a regular culture medium enriched with the DDS and CBD treatments, respectively. The working concentration ranges for DDS (6.04–30.6 µM) and CBD (0.047–0.24 µM) were determined following the cytotoxicity study detailed in the preceding paragraphs. The greatest concentration of each agent found to maintain 90% cell viability was used to determine these ranges. The plates supplemented with the treatments were incubated for 24 h in normoxic and glucose-containing culture media. After this period, the MTT method was used to reveal cell viability and expressed as a percentage of untreated cell viability. The cytoprotection index was computed using the viability percentage values, employing the following formula:

Cytoprotection%=viability%treatment−Viability%OGD/R

The effective cytoprotective concentration of 50 (EC_50_) of each component, EC_50_-DDS and EC_50_-CBD, was computed using concentration–effect relationship curves and logit–probit analysis [19].

### 2.6. Assessment of the Effect of DDS–CBD Combination Using Isobolographic Analysis

An isobolographic analysis [20] was performed to investigate the pharmacological interactions between DDS and CBD. According to Miranda et al. [21], combinations of fixed proportions were made with the EC_50_-DDS and EC_50_-CBD values, and the culture medium was supplemented with the following mixture of DDS–CBD at proportions: 1:1, 1:4, and 4:1, respectively. Mixture concentrations were determined using the following formulas for each drug: 1:1, EC_50_ = 0.5 (EC_50_ DDS) + (1 − 0.5) (EC_50_ CBD); 1:4, EC_50_ = 0.25 (CE_50_ DDS) + (1 − 0.25) (EC_50_ CBD); 4:1, CE_50_ = 0.75 (EC_50_ DDS) + (1 − 0.75) (EC_50_ CBD). All treatments were dissolved in 0.01% DMSO mixes. According to the methodology stated at the beginning of the section, differentiated SH-SH5Y cells were exposed to OGD/R culture conditions for 6 h before being replaced with glucose-rich culture media containing the appropriate DDS–CBD treatment mixes and controls, respectively. Under standard conditions, the plate was incubated for 24 h. Finally, using the MTT method, the percentage of cell viability was calculated by contrasting cells subjected to culture standard conditions. Using the cell viability data, under OGD/R culture conditions, the mixing ratio that was significantly more protective was chosen.

In order to calculate the mean experimental effective concentration (EC_50_E) that protects SH-5HSY cell viability from OGD/R conditions, the range of concentrations required a second OGD/R experiment that was designed around the DDS/CBD mixture ratio that resulted in the highest percentage of cell viability. To determine the concentrations of the working solutions, values were established upstream and downstream of the most effective mixing concentration, excluding this same value from the assay. All treatment solutions were prepared with a culture medium enriched with the compounds of each concentration previously dissolved in 0.01% DMSO. As previously mentioned, the MTT technique was employed to assess cell viability. Subsequently, the percentage viability was determined by comparing the results to a control group of cells from the identical subculture subjected to standard conditions for 30 h.

Based on the assumption that the interaction between the two substances was additive, a simple graph was created depicting the ratio of DDS to CBD equieffective concentration ratios [22]. This was performed using Cartesian coordinates to place EC_50_-DDS (value ± SEM) on the y-axis and EC_50_-CBD (value ± SEM) on the x-axis. An isobola (straight line) was created from the two concentrations. The DDS-to-CBD-concentration ratios given below are those that should result in the average cytoprotective effect if the additive-type interaction between DDS and CBD occurs.

The method below can be used to calculate the additivity index (i) for any given point in this interaction, as well as the precise values of its DDS and CBD components [23]:

i = C1-DDS/EC_50_-DDS + C2-CBD/EC_50_-CBD (1)

Which represents the concentrations of DDS and CBD that produce the desired effect when taken together; in this case, they are represented by C1-DDS and C2-CBD, respectively, while EC_50_-DDS and EC_50_-CBD represent the corresponding values of the mean effective individual cytoprotective concentrations of the DDS and CBD, respectively. To find the relevant concentrations, the following formula was used to compute the corresponding EC_50_-DDS and EC_50_-CBD fractions:

1 = f-DDS + f-CBD(2)

where f-DDS and f-CBD are the fractions of DDS and CBD, respectively. Finally, the interaction index was calculated using the formula

I = EC_50_E/EC_50_T (3)

Theoretical effective concentration (EC_50_T) is derived from the above equations with the experimental values of the EC_50_ of individual effects of DDS and CBD, respectively, and EC_50_E is obtained directly from the concentration–effect curve of the most effective mixture. The interaction indices of the experimental combination (EC_50_E) and the theoretical (EC_50_T) were then compared using Student’s *t*-test, with *p* < 0.05 indicating a significant difference. Tallarida [24] defines a synergistic (sub-additive) interaction as I < 1.0, additive (I = 1.0), or antagonistic (supra-additive) interaction as I > 1.0.

Finally, to determine the parameters for evaluating the pharmacological and biochemical activities of the DDS–CBD mixture, the EC_90_E obtained from the concentration-effect curve mentioned above was used.

### 2.7. Lactate Dehydrogenase (LDH) Assay

Cytotoxicity was assessed through the release of LDH [25]. SH-SY5Y cells from different groups were cultured in 96-well plates (4 × 10^3^ cells/well), subjected to oxygen–glucose deprivation for 6 h, and reoxygenation (OGD/R) for 24 h with pharmacological treatment. LDH released from cells was detected using the LDH assay kit (11644793001, Roche). The supernatant (100 μL) and reaction mix (100 μL) were mixed in 96-well plates and incubated for 1 h at 37 °C. The absorbance was measured at 490 nm using a microplate spectrophotometer (BioTek Eon™). Results were expressed as a percentage of control group cells. All experiments were performed in nine replicates per group. The LDH release rate was calculated with the following formula:

LDH cytotoxicity (%) = [OD (treatment) − OD (blank)]/[OD (maximum) − OD (blank)] × 100%.

### 2.8. Measurement of Intracellular ROS

5-(and-6)-chloromethyl-2′,7′-dichlorodihydrofluorescein diacetate (CM-H2DCFDA) is an indicator of general oxidative stress used to measure the level of intracellular ROS production. Seeding 96-well plates with 1 × 10^3^ cells/well for 24 h promotes cell adhesion. After being washed twice in Hank’s medium, the cells were exposed to OGD for 6 h before being reoxygenated with 100 mL of the alternative test treatments for 24 h. Following exposure, cells were washed twice with Hank’s solution and incubated for 1 h in the dark in an incomplete culture medium (0.02 M CM-H2DCFDA) (medium without FBS supplement). A Varioskan lux multi-plate reader (Thermo Fisher Scientific, Inc., Waltham, MA, USA) was used at 485 nm for excitation and 528 nm for emission to measure the fluorescence intensity.

### 2.9. Measurement of Reduced Glutathione (GSH)

After seeding SH-SY5Y cells in 6-well plates and treating them with OGD/R conditions, the cells were harvested and sonicated in SSF at cold temperatures, 25% metaphosphoric acid was added to the sample in a 1:1 ratio, and the mixture was centrifuged at 13,000 rpm for 15 min at 4 °C. The supernatant was then taken, and EDTA buffer and the O-phthalaldehyde indicator were added. It was incubated at room temperature for 15 min with a 1 mg/mL concentration. The fluorescence intensity was measured using a Varioskan lux multi-plate reader (Thermo Fisher Scientific, Inc., Waltham, MA, USA) with an excitation wave of 350 nm and an emission wave of 420 nm. GSH (5 mg/mL) generated a standard curve.

### 2.10. Caspase-3 Activity Assay

Cellular apoptosis was assessed with a caspase-3 colorimetric kit (APT165, Merck Millipore) that identifies the DEVD amino acid sequence. A concentration of 1 × 10^6^ cells/mL was placed in a 6-well plate, and they were subjected to oxygen–glucose deprivation for 6 h and reperfusion (OGD/R) for 24 h with drug treatment divided into different groups. The cells were recovered by scraping and centrifuged at 1500 rpm for 10 min; the supernatant was removed, and the button was reconstituted with 1× buffer for 5 min. The supernatant was taken and placed in a 96-well plate. Finally, 5× assay buffer and caspase-3 substrate were added; they were incubated at 37 °C for 1 h. A standard curve of p-nitroanilide (pNA) in a concentration range of 10 μM to 1 mM was generated by diluting the pNA stock solution in 1× assay buffer. Then, 100 μL of each dilution was placed in the ELISA plate, and they were read together with the samples in a fluorescence spectrophotometer at 405 nm using a microplate spectrophotometer (BioTek Eon™, Winooski, VT, USA). The results were expressed as a percentage of control group cells. All experiments were performed in nine replicates per group. Caspase-3 activity was expressed as a percentage compared to the control group.

### 2.11. Statistical Analysis

In all cases, exploratory data analysis was performed to determine whether there was a normal distribution, applying the Kolmogorov–Smirnov test and homogeneity of standard error, applying the Levene test. Once this was determined, parametric statistical tests such as one-way ANOVA, followed by Dunnett post hoc test and mortality in SH-SY5Y cells analysis, LC_50_ and EC_50_ values (with 95% confidence limits) were determined by probit regression analyses. Data were analyzed using SPSS, version 25.0 software. The results of this study were performed in nine replicates per group and were expressed as means ± SEM with a significance level of * *p* < 0.05, ** *p* < 0.01, *** *p* < 0.001.

## 3. Results

### 3.1. Cytotoxicity Assay of Individual Drugs

**Figure 1:**
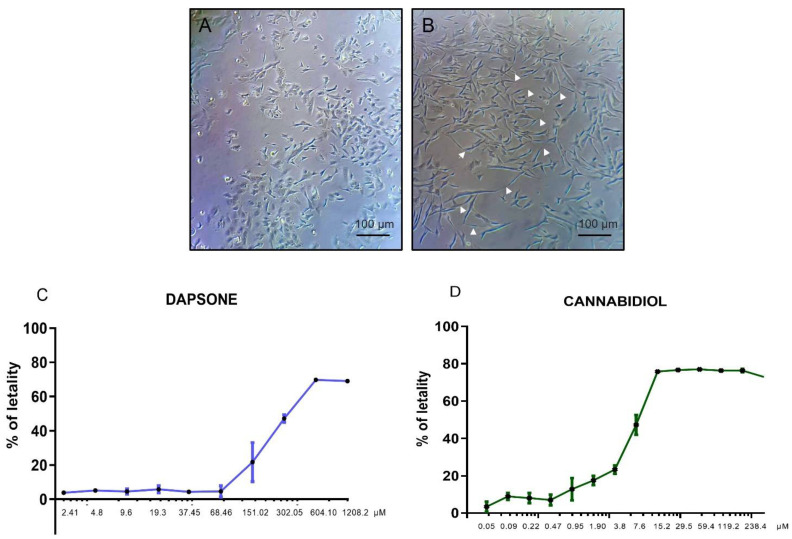
Percentage of lethality in SH-SY5Y cells after exposure to DDS or CBD. (**A**) Morphological appearance of undifferentiated SH-SY5Y cells where a flat surface with few projections are observed, while (**B**) differentiated SH-SY5Y neurons demonstrate extensive and elongated neuritic projections (white arrow). Images were obtained in phase at 20× magnification using an inverted epifluorescence microscope. Percentage of lethality in differentiated SH-SY5Y cells after exposure to DDS (**C**) or CBD (**D**) at different concentrations for 24 h. Values are expressed as percentages compared to the control group and are the mean ± SEM of the mean of three replicates per group. Probit regression analysis was performed.

The results of the cytotoxicity effect are shown in Figure 1 for DDS and CBD. The viability of SH-SY5Y cells decreased in a concentration-dependent manner when exposed to the drugs individually. CBD had a cytotoxic threshold of 0.95 µM compared to DDS’s 151.0 µM. Furthermore, DDS exhibited a lethal concentration 50 (LC_50_) of 412.8 µM, whereas CBD demonstrated an LC_50_ of 12.9 µM.

### 3.2. Cytoprotective Effect of Dapsone and CBD in a Model Oxygen Glucose Deprivation/Reoxygenation in SH-SY5Y Cells

**Figure 2:**
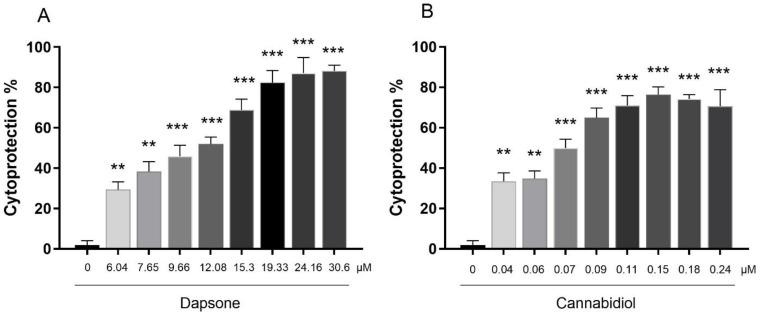
Panel (**A**), the effect of dapsone (DDS) and cannabidiol (Panel (**B**)) evaluated in an oxygen–glucose deprivation and reoxygenation (OGD/R) model in SH-SY5Y cells. The bars represent the average value expressed in percentage ± SEM of nine replicates per group. One-way ANOVA followed Dunnett’s test. ** *p* < 0.01, *** *p* < 0.001 vs. control group.

In this section, the cytotoxic threshold values from the previous experiment were utilized to determine the concentrations of DDS (6.04, 7.65, 9.66, 12.08, 15.30, 19.33, 24.16, and 30.6 µM) and CBD (0,04, 0.06, 0.07, 0.09, 0.11, 0.15, 0.18, and 0.24 µM) to assess the cytoprotective effect separately of both in the OGD/R model. The viability of the cells was significantly higher in the group pretreated with 30.6 µM DDS, showing an 88.18 ± 1.6% response compared to the OGD/R without treatment, as shown in Figure 2 panel A, while CBD had its most significant effect at 0.15 µM concentration, which shows 76.35 ± 2.2% cytoprotection when compared to the OGD/R group without treatment (Figure 2, panel B). The effective cytoprotective concentration 50 (EC_50_) of DDS and CBD obtained were 10.06 ± 0.22 µM and 0.07 ± 0.004 µM, respectively.

### 3.3. Effect of DDS–CBD Combination by the Isobologram Method

**Figure 3:**
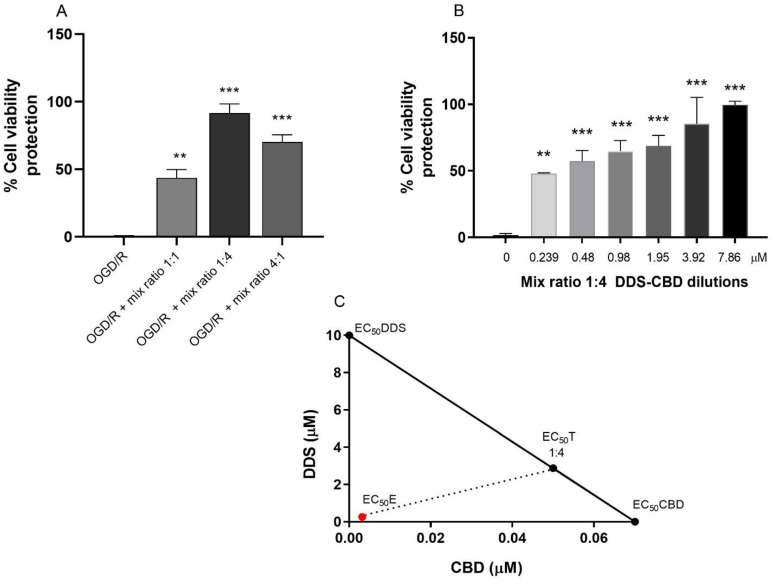
Isobolographic analysis of the DDS–CBD combination. Effect of the combination of dapsone and cannabidiol at three different ratios (1:1, 1:4, and 4:1) on cell viability (Panel (**A**)) in an OGD/R model. Effect of the combination of DDS–CBD in a 1:4 ratio at different dilutions (Panel (**B**)). Isobologram of the drug interaction between DDS–CBD (Panel (**C**)) on cell viability in OGD/R model with SH-SY5Y cells. The plotted additivity line shows the EC_50_ of the individual drugs; the dots (●) correspond to the theoretical EC_50_T of the 1:4 ratio. Similarly, the corresponding value of the experimental EC_50_E of the 1:4 ratio (●). An interaction index (I = EC_50_E/EC_50_T) of 0.10 was obtained, establishing a synergistic effect. The bars represent the average value expressed in percentage ± SEM of nine replicates per group. One-way ANOVA followed Dunnett’s test. ** *p* < 0.01, *** *p* < 0.001 vs. control group.

Table 1 provides a summary of the calculated concentrations of the components of the DDS–CBD mixture in each of the evaluated proportions, as determined by the EC_50_ value of each agent [26]. The exposure to all combinations significantly increased cell viability in SH-SY5Y cells under oxygen–glucose deprivation and reoxygenation (OGD/R). However, the combination with a 1:4 mix ratio showed the maximum effect on the other combinations, where an effect of 91.62% cell viability recovery response was observed (Figure 3, panel A).

The range of dilutions for the 1:4 drug mixture was determined by the cytoprotective activity of 91.62% of the 1:4 ratio, which had a final concentration of 2.55 µM (Table 1). An independent assay was conducted to determine the experimental 50% effective concentration (EC_50_E) of the 1:4 ratio of DDS–CBD, resulting in 0.26 µM, as shown in Figure 3 panel B.

The value of the theoretical 50% effective concentration of the 1:4 ratio mixture, as determined by the formula provided in Table 1, was 2.55 µM, shown by the isobologram in panel C of Figure 3. As stated in Table 2, a comparison of the EC_50_T and EC_50_E values revealed a significant difference of *p* < 0.05 and an interaction index of 0.10.

### 3.4. DDS–CBD Treatment Improves Cell Viability and Attenuates Oxidative Damage

**Figure 4:**
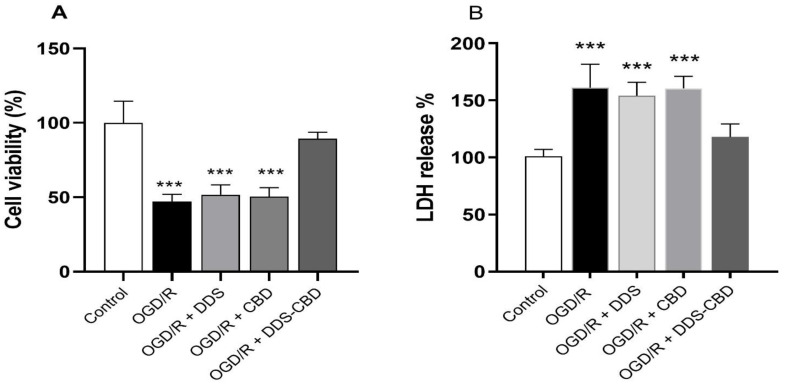
Effect of DDS (7.55 µM) and CBD (0.311 µM) alone or combined (DDS + CBD 7.86 µM), corresponding to the effective concentration 90 (EC_90_) on cell viability (Panel (**A**)) and LDH release (Panel (**B**)) in SH-SY5Y cells with oxygen–glucose deprivation/reoxygenation. The bars represent the average value expressed as a percentage ± SEM of nine replicates per group. One-way ANOVA followed Dunnett’s test. *** *p* < 0.001 vs. control group.

Figure 4, Panel A shows the viability of SH-SY5Y cells subjected to oxygen and glucose deprivation (OGD/R), where it is decreased in those cells that did not receive treatment (47.19 ± 1.58). The groups treated with DDS (51.69 ± 2.21) and CBD (50.38 ± 2.01) do not show a statistically significant difference compared to the group subjected to conditions of OGD/R. In contrast, the group treated with the DDS–CBD (89.37 ± 1.44) mixture presents a statistically significant difference vs. group OGD/R.

Similarly, the measurement of cellular LDH release revealed the conditions of cells’ cytotoxicity. The result demonstrated that the DDS–CBD combination is an efficient treatment against cellular oxidative damage because the group treated with this mixture at a total concentration of 7.86 µM (7.55–0.311 µM) showed a significant decrease in the release of LDH (17.86% ± 3.31) compared to the group subjected to conditions of oxygen–glucose deprivation without treatment (OGD/R) where an increase of 61.22% ± 5.9 vs. control group is observed, while the OGD/R-DDS and OGD/R-CBD groups do not show a statistically significant difference (*p <* 0.05) compared to the OGD/R group as shown in Figure 4, panel B.

### 3.5. DDS–CBD Combination Decreased Oxidative Stress Induced by OGD/R

**Figure 5:**
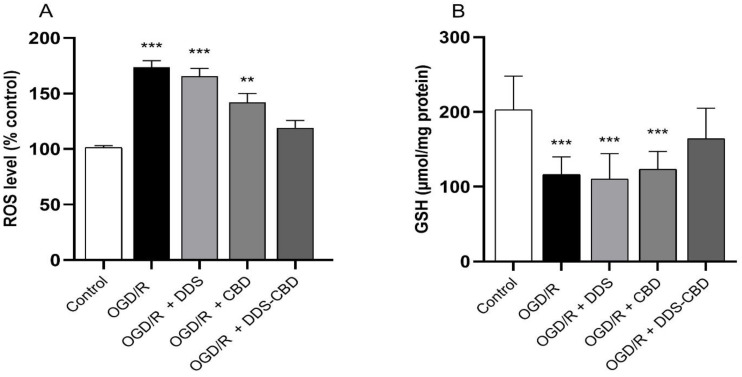
Effect of treatment with the DDS–CBD combination upon reactive oxygen species (ROS) production (Panel (**A**)) and reduced glutathione (GSH) levels (Panel (**B**)) on SH-SY5Y cells subjected to OGD/R. The values are expressed as a percentage vs. control group and are the average ± SEM of nine replicates per group and were analyzed by one-way ANOVA followed by Dunnett’s test. ** *p* < 0.01, *** *p* < 0.001 vs. control group.

The effect of the DDS–CBD combination on the oxidative stress induced by OGD/R was evaluated by detecting levels of ROS and GSH. Figure 5 (Panel A) shows a considerable rise in ROS levels in the OGD/R group (73.7%) vs. the control group (173.7 ± 5.8). In comparison, the group treated with the DDS–CBD combination increased only by 19% vs. the control group (119.01 ± 6.72), indicating a considerable reduction in ROS levels. The damage caused by OGD/R significantly decreased GSH levels (Figure 5 Panel B) (116.45 ± 7.82). On the contrary, when the cells were treated with DDS–CBD (164.21 ± 13.6), the GSH levels were increased compared to the OGD/R group, which showed a statistically significant difference, while the drugs separately showed no difference with the OGD/R group.

### 3.6. DDS–CBD Treatment Decreased OGD/R-Induced Caspase-3 Activity

**Figure 6:**
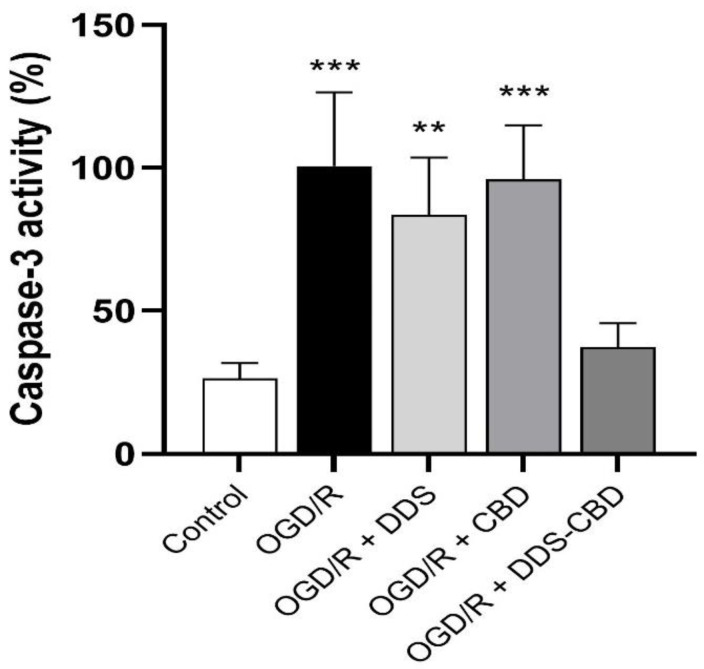
Caspase-3 activity induced by OGD/R on SH-SY5Y cells. The bars represent the average value expressed as a percentage ± SEM of nine replicates per group and were analyzed by one-way ANOVA followed by Dunnett’s test. ** *p* < 0.01, *** *p* < 0.001 vs. control group.

Figure 6 shows the results of exposure to the DDS–CBD mixture 24 h after oxygen–glucose deprivation on caspase-3 enzymatic activity. The results show that under normoxia conditions (26.45 ± 1.77), caspase-3 activity is at low levels compared to the group subjected to OGD/R conditions without treatment (100.68 ± 8.58). On the other hand, in the group treated with DDS (67.92 ± 3.29) and CBD (56.89 ± 5.91), no significant differences were observed vs. OGD/R group values since CBD increased the enzymatic activity of caspase-3 compared to the group under normoxia conditions by 30.2%, while the group treated with the DDS–CBD mixture (37.25 ± 2.81) only increased 10.8% of the enzymatic activity of caspase-3.

## 4. Discussion

**Figure 7:**
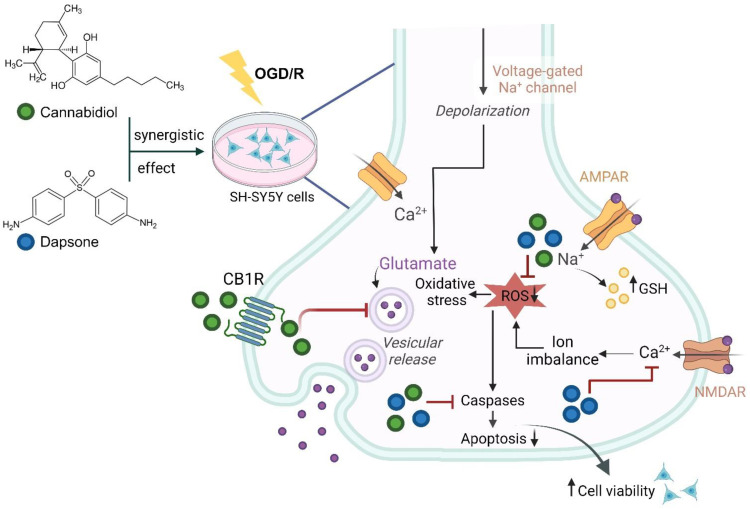
Synergistic effect of dapsone and cannabidiol on the damage caused by OGD/R. Cerebral ischemia is characterized by the interruption of blood flow, which in turn decreases oxygen and glucose in neurons, which leads to neuronal excitotoxicity, triggering a cascade of processes leading to the production of ROS, an imbalance of ions, activation of the caspases pathway and cell death by apoptosis. Cannabidiol acts on CB1 receptors, thus regulating the release of glutamate in pre-synaptic neurons, while dapsone acts on postsynaptic neurons, regulating the excessive release of calcium and ROS, thus modulating the activation of caspases. Oxygen–glucose deprivation/reoxygenation (OGD/R), cannabinoid receptor 1 (CBR1), α-amino-3-hydroxy-5-methyl-4-isoxazolepropionic acid receptor (AMPAR), reactive oxygen species (ROS), N-methyl-D-aspartate (NMDAR).

Several studies have pointed out the beneficial effects of CBD, showing that it has an antioxidant and anti-inflammatory effect on different brain pathologies such as epilepsy, Parkinson’s disease, and anxiety in different models, including in vitro experiments [14,27,28]. On the other hand, DDS has been described as a neuroprotective agent in rat focal ischemia by us and other authors [29,30]. Due to the significant impact of cerebral ischemia on neurons, the present study evaluates the effect of the combination of CBD and DDS as an antioxidant and antiapoptotic drug in an in vitro model of SH-SY5Y human neuroblastoma cells; these cells not only provide a model of cerebral ischemia in cell culture but also possess essential characteristics for this study since SH-SY5Y neuronal cells have been shown to selectively express the CB1 receptor [31,32], in addition, retinoic acid-induced neuronal differentiation was associated with a substantial increase in dopamine receptor subtypes (D2R and D3R) as well as dopamine transporter expression [33,34,35]. To know the activity resulting from the association of the two molecules, an isobolographic study was carried out, in which it was observed that the combination of DDS and CBD has a synergistic effect in the model of cerebral ischemia with reperfusion in SH-SY5Y cells, protecting the cells. The synergistic effect of these two drugs demonstrates a 10-fold reduction in the individual equipotent concentration of DDS and CBD, implying that the protective effect of the mixture is achieved with less risk of potential toxic effects. However, the clinical significance of this will need to be elucidated with further studies. CBD demonstrates strong antioxidant and anti-inflammatory activity in various experimental paradigms, an effect that has been linked to the activation of CB1 and CB2 receptors [36], because a decrease in ATP causes the membrane potential to be lost, resulting in neuronal depolarization and the release of large amounts of glutamate in the early stages of ischemia. CBD has been shown in previous research to inhibit calcium flow across membranes, stabilize mitochondrial membranes, modulate many types of receptors, including serotonin 5HT1A and peroxisome proliferator-activated receptor gamma (PPARγ) receptors, increase extracellular activity, reduce adenosine concentration, and inhibit nuclear factor NFκB activation [37,38,39]. It has also been reported that elevated levels of anandamide, the main component of the endocannabinoid system, can activate CB1 receptors by reducing glutamate release because CBD inhibits the degradation of anandamide enzyme, decreasing the excitotoxicity that is triggered by the excessive release of glutamate [40,41,42]. In addition to the direct neurotoxicity of glutamate in neurons, the activation of glutamate receptors subtypes: N-methyl-D-aspartate (NMDA-) and α-amino-3-hydroxy-5-methyl-4-isoxazolepropionic acid receptor (AMPA-); leads to a further increase in intracellular Ca^2+^ levels, making dapsone a drug capable of decreasing the flux (Figure 7) of this ion since it is an antagonist in calcium-dependent processes, preventing the activation of metabotropic and ionotropic receptors (m-GluR and NMDA, respectively), as well as Glu receptors (AMPARs) and kainate (KA), which leads to preventing membrane depolarization by inhibiting the opening of voltage-gated calcium channels (VGCC), voltage-gated sodium channels (VGSC), and voltage-gated potassium channels (VGKC), which regulate the signaling cascade and ion release, as reported by Suda et al. [43]. Dapsone has also been shown to have an antioxidant effect since it has been observed that it decreases the formation of superoxide radical (O_2_^−^) and hydrogen peroxide (H_2_O_2_) produced in the mitochondria during the excitotoxic and ischemic process, reducing the production of NO and peroxynitrite (ONOO-). On the other hand, a study of cerebral ischemia in rats showed that the administration of DDS regulates both the intrinsic and extrinsic pathways of apoptosis mediated by the activation of several caspases, preventing cell death [44,45,46]. All of these actions explain DDS–CBD’s potential neuroprotective effectiveness in the OGD/R model, as seen in Figure 7.

Cellular ischemia is characterized by altered cellular markers, including decreased cell viability, increased LDH release, increased ROS production, and decreased GSH activity. Our findings confirmed that DDS–CBD treatment could protect cells from oxidative stress by counteracting these markers. These findings are consistent with a study that found CBD had a protective effect at low concentrations against oxidative stress induced by H_2_O_2_ [47,48,49], suggesting that CBD protects PC12 cells from damage caused by exposure to β-amyloid protein, preserving cell viability while reducing ROS and lipid peroxidation. Castillo et al. [50] reported that CBD administration reduced LDH release and caspase-9 expression in an in vitro brain cell model of newborn mice exposed to OGD, indicating a decrease in cell death from both necrotic and apoptotic processes. In terms of dapsone’s neuroprotective impact, several studies demonstrate that it has antioxidant properties since it inhibits oxidative stress indicators such as ROS production and lipoperoxidation [51,52]. Wozeland Blasum [11] showed that DDS can scavenge hydroxyl radicals and reduce hydrogen peroxide concentrations due to the oxidative activation of polymorphonuclear leukocytes, decreasing GSH levels. As described above, DDS–CBD treatment prior to exposure to the pro-oxidant agent restored GSH levels to normal values in SH-SY5Y cells studied here.

Calcium dysregulation, increased glutamatergic signaling, oxidative stress, and inflammatory responses are critical factors triggering cell apoptosis in cerebral ischemia [53,54,55]. After an ischemic event, genetic overexpression of the enzyme caspase-3 has been observed [56,57]. Our findings demonstrated that caspase-3 activity decreased in SH-SY5Y cells under OGD/R conditions treated with DDS–CBD, showing a decrease in apoptosis. This event is consistent with current findings where reduced or null expression of caspase-9 and caspase-3 as observed in an in vitro model of cerebral ischemia, Alzheimer’s [50,58,59], as well as cells treated with hydrogen peroxide [60], after exposure to CBD. On the other hand, it has also been observed that DDS is an antiapoptotic drug, as revealed in previous studies in which the administration of DDS decreased the activities of caspase-9 and caspase-3 in models of focal cerebral ischemia [29] and spinal cord injury in rats [61].

## 5. Conclusions

With all this evidence, it is the first time that it has been demonstrated that the combination of the drugs dapsone and cannabidiol have a synergistic effect, enhancing the effect of each of the drugs, in addition to the fact that it was observed that the treatment protected SH-SY5Y cells, reducing oxidative stress and cell death by apoptosis after being subjected to oxygen–glucose deprivation and reperfusion. These results suggest that the DDS–CBD combination may be a potential therapeutic candidate to prevent or slow neuronal degeneration after an ischemic cerebrovascular event.

## Figures and Tables

**Table 1:** Dapsone–Cannabidiol ratios to evaluate the mixture in the OGD/R model.

Proportions	Formula	DDS (µM)	CBD (µM)	Final Combination (µM)
1:1	EC_50_ = 0.5 (EC_50_ DDS) + (1 − 0.5) (EC_50_ CBD)	5.03	0.035	5.065
1:4	EC_50_ = 0.25 (EC_50_ DDS) + (1 − 0.25) (EC_50_ CBD)	2.5	0.05	2.55
4:1	EC_50_ = 0.75 (EC_50_ DDS) + (1 − 0.75) (EC_50_ CBD)	7.54	0.01	7.55

Oxygen–Glucose Deprivation/Reoxygenation (OGD/R), Dapsone (DDS), Cannabidiol (CBD), EC (Effective concentration).

**Table 2:** EC50 ± SEM (µM) and interaction index for the isobolographic analysis of dapsone with cannabidiol in oxygen-glucose deprivation/reoxygenation model in SH-SY5Y cells.

EC_50_ ± SEM			
Combination	Theoretical	Experimental	Interaction Index
1:4 ratio	2.55 ± 0.013	0.26 ± 0.039	0.10 *

* *p* < 0.05 comparing EC_50_ theoretical with EC_50_ experimental, Student’s *t*-test.

## Data Availability

The following repository offers access to study data set: Islas Cortez, Marcela; Rios, Camilo; Manzanares, Jorge; Diaz-Ruiz, Araceli; Perez Pasten Borja, Ricardo (2024), “DATA SET: Isobolographic analysis of the cytoprotective effect of dapsone and cannabidiol alone or combination upon oxygen-glucose deprivation/reoxygenation model in SH-SY5Y cells”. Mendeley Data, V1, doi: 10.17632/3ctsn9ww67.1
